# Effects of Current on the Membrane and Boundary Layer Selectivity in Electrochemical Systems Designed for Nutrient Recovery

**DOI:** 10.1021/acssuschemeng.2c01764

**Published:** 2022-07-15

**Authors:** Mariana Rodrigues, Tom Sleutels, Philipp Kuntke, Cees J. N. Buisman, Hubertus V. M. Hamelers

**Affiliations:** † Wetsus, European Centre of Excellence for Sustainable Water Technology, Oostergoweg 9, 8911MA Leeuwarden; P.O. Box 1113, 8900CC Leeuwardem, The Netherlands; ‡ Environmental Technology, Wageningen University, Bornse Weilanden 9, 6708 Wageningen; P.O. Box 17, 6700 AA Wageningen, The Netherlands

**Keywords:** limiting current, dynamic state, electrochemical systems, boundary layer−membrane selectivity, nutrient recovery

## Abstract

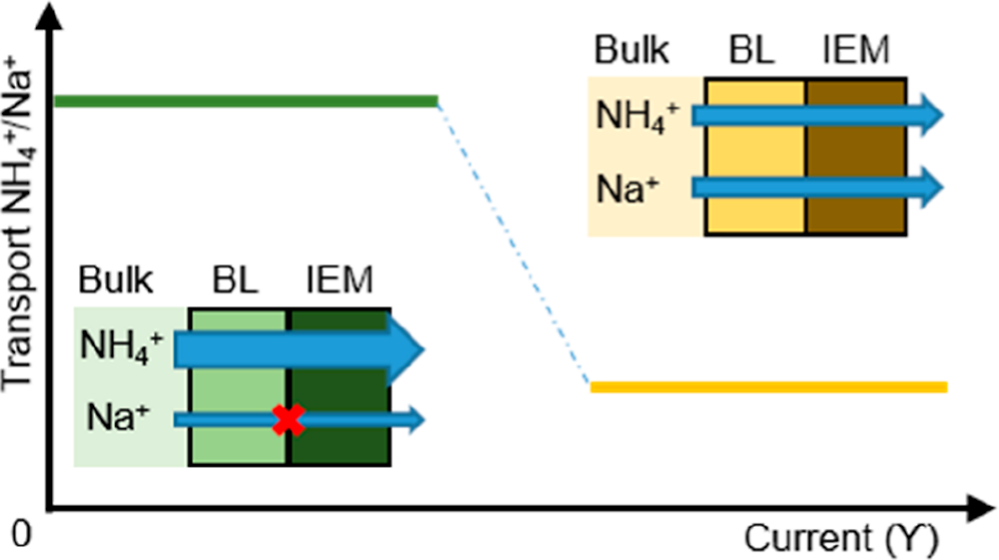

During electrochemical nutrient recovery, current and ion exchange membranes (IEM) are used to extract an ionic species of interest (e.g., ion) from a mixture of multiple ions. The species of interest (ion 1) has an opposing charge to the IEM. When ion 1 is extracted from the solution, the species fractions at the membrane and the adjunct boundary layers are affected. Hence, the species transport through the electrochemical system (ES) can no longer be described as electrodialysis-like. A dynamic state is observed in the compartments, where the ionic species are recovered. When the boundary layer–membrane interface is depleted, the IEM is at maximum current. If the ES is operated at a current higher than the maximum current, the fluxes of both ion 1 and other competing ions, with the same charge (ion 2), occur. This means, for example, ion 1 will be recovered, and the concentration of ion 2 will build up in time. Therefore, a steady state is never reached. Ideally, to prevent the effect of limiting current at the boundary layer–membrane interface, ES for nutrient recovery should be operated at low currents.

## Abstract 2

We should consider both the selectivity of the IEM and the boundary layer to operate membrane electrolysis systems for nutrient recovery more efficiently.

## Introduction

Over the last few decades, electrochemical systems (ES) have been developed for diverse fields, for the production of raw materials, energy production, and nutrient recovery.^1−3^ This diverse use of ES has led to several studies on, among others, the electrode reactions, chemical efficiency, membrane resistance, and ion transport over the membrane.^4−9^

An ES is a system including a minimum of two electrodes immersed in a solution and connected through an electric circuit. Typically at the electrodes, reduction and oxidation reactions occur when current is applied. Consequently, electrons move through the external circuit, and charged species move in the solution accordingly to balance the charge in the system (maintain electro neutrality). The introduction of ion exchange membranes (IEMs) in ESs allowed for membrane electrolysis and consequently the separation of charged molecules (i.e., ions) from the electrolyte or wastewater.^10−13^ Additionally, as the IEMs block specific ions (coions), undesired reactions at the electrodes are prevented (e.g., chlorine gas formation at the anode).

In membrane electrolysis, counter-ions migrate through the IEMs due to the current, leading to the depletion of ions in one solution and enrichment in another solution. Three distinct operation regimes can be described for IEMs as a function of the current (density); (i) ohmic, (ii) limiting, and (iii) overlimiting current region.^14−16^ In the ohmic region, increasing current density directly increases the charge transported per membrane area. An even higher current density will lead to a depletion of ions in the boundary layer. In this case, the ion concentration is close to zero on one side of the IEM, and the resistance increases. Here, the system operates at limiting current density, decreasing current efficiency for the desired ion transport. If we increase the current density further, the system operates at overlimiting current, and the conventional laws do not apply. A general limiting current density is often pre-established by the membrane manufacturer, using only known solutions of NaCl and a constant gradient between compartments.^17−19^

Depending on the application, IEMs can be operated at different current densities, 0.1 A m^–2^ up to 1000 A m^–2^.^9,19,20^ Often, the current density is increased to improve the flux of ions (transport rate) over the IEM and therefore increase the treatment capacity. However, when increasing the current density while maintaining the amount of ions loaded to the system, the current efficiency will decrease as the system is in the limiting current regime.^21,22^

**Figure 1:**
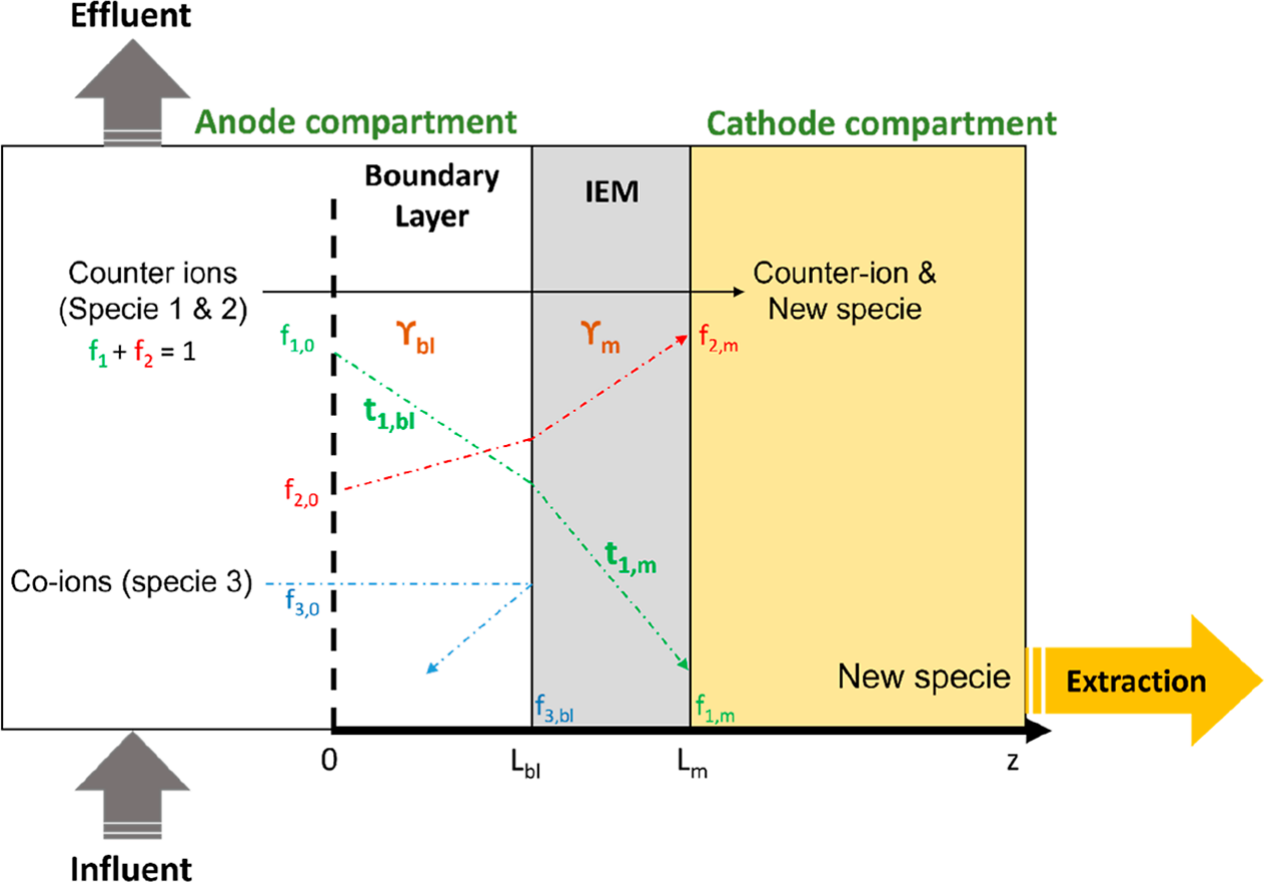
Scheme describing membrane electrolysis for ion recovery. Wastewater is supplied to an electrochemical system, including an ion exchange membrane (IEM) separating the anolyte from the catholyte. Counter-ions are transported over an IEM due to the applied current (γ). A dimensionless current density (flux) is attributed to each region, boundary layer (γ_bl_), and membrane (γ_m_). Two counter-ions are supplied to the anode, and the sum of their fraction equals one. Once transported, ion 1 reacts and forms a new species, and ion 2 accumulates at the cathode. The new species is removed from the cathode due to an additional extraction process. Ion 3 represents all anions. The fraction of the different species (*f*) is characterized at the anolyte–boundary layer interface (0), boundary layer–membrane interface (*L*_bl_), and at the membrane–catholyte interface (*L*_m_). The relation between fraction (*f*_1_), transport (*t*_1_), and current (Υ) will be established for ion 1.

When membrane electrolysis is implemented in, for example, resource recovery, the inclusion of membranes, the supply of different streams with extreme pH or variable compositions, or the need to operate in continuous mode should be investigated.^11,22−24^ Furthermore, the pairing of membrane electrolysis with efficient extraction processes such as precipitation or stripping (i.e., gas-permeable membranes) creates dynamic conditions within the system, as represented in Figure 1.^23,25−27^ This can cause low efficiency, limited ion transport, and high energy consumption. For example, during electrochemical ammonia recovery from wastewater, the transport of ammonium over the IEM is preferred (ammonium transport number equals one). However, it was previously observed that all cations in solution are transported through the cation exchange membrane (CEM) toward the cathode. While NH_3_ is removed from the cathode by a gas-permeable membrane, the other cations accumulate in the catholyte.^28−30^ The transport of other undesired charged species over the CEM lowers the current efficiency. Additionally, the catholyte does not reach equilibrium, influencing ion transport over the CEM, separating the anolyte from the catholyte. Here, the selectivity of the IEM results in different ion transport (behavior) than during electrodialysis.^12,31,32,33^

In this work, we explore why more than one ionic specie is transported over an ion exchange membrane in some electrochemical systems, while the recovery process only recovers one species of interest. A simplified model was used to study how the ion fractions influence the ion transport over the boundary layer and CEM when increasing the current (density). We will also show that the ionic species transport over the boundary layer–membrane ensemble is affected by both the current (density) and anolyte (feed) composition and describe how we can maximize the transport (flux and efficiency) of the desired ion that is to be recovered from the catholyte.

## Theoretical Framework

This work describes the dynamic behavior of a quasi-steady-state membrane electrolysis system. Here, the system consists of a well-mixed anode compartment (with only monovalent ions), a boundary layer, an ideal IEM (meaning only counter-ions are transported through the membrane, as coion leakage over CEMs is very low compared to the total charge supplied; and the ions have equal mobility in the membrane, as their mobility, self-diffusion coefficients, and ionic Stokes radii are the same order of magnitude^18,101,102^), a well-mixed catholyte, and an efficient extraction process at the cathode (Figure 1). The anolyte solution only includes monovalent ions (i.e., NH_4_^+^, Na^+^, and Cl^–^). As the pH of the catholyte is alkaline, the presence of bivalent cations would either cause inorganic scaling or require a pretreatment, and the transport over the membrane could not be characterized the same way.^103,104^ The catholyte includes both counter-ions transported (i.e., NH_4_^+^ and Na^+^). The hydroxyl ions (OH^–^), generated during the electrode reaction, react with NH_4_^+^ and form a new species (i.e., NH_3(aq)_ ↔ NH_4_^+^ + OH^–^). The protons generated by the anode reaction will not be included in the model, as they behave similarly to NH_4_^+^. Protons are also transported through the CEM and react with the OH^–^ at the catholyte. The influent is supplied continuously to the anode compartment, generating a treated effluent. On the other side of the IEM, a finite volume is recirculated in batch (catholyte). The concentrated solution formed at the cathode is recirculated over an extraction process, wherein this new species (NH_3_) is recovered from the solution. The system described represents any system, where current is applied; at least two cationic species (i.e., NH_4_^+^, Na^+^) are supplied to the anode compartment and transported to the cathode compartment through the CEM. In a second process, NH_3_ is removed from the catholyte by a different process (e.g., NH_3_ stripping, membrane extraction, etc.).

To reduce the operation variables, we will present all parameters dimensionless.

### Dimensionless Nernst–Planck Equation

When current is applied, the transport of ions is driven by the concentration gradient over the membrane and the potential gradient between the anode and cathode. This transport is commonly described by the Nernst–Planck (NP) equation. We considered three main species: ion 1, NH_4_^+^, either recovered or consumed at the cathode side. Ion 2, Na^+^ represents the sum of all other cations accumulated at the cathode side. Ion 3, Cl^–^ represents the sum of all anions. Additionally, T^+^ is the sum of all cations (NH_4_^+^ and Na^+^) and T^–^ is the sum of all anions (in this case, Cl^–^). In the considered system, the ions are supplied to the anode compartment, where a boundary layer near the CEM is formed due to applied current. The counter-ions move through the boundary layer, into the IEM, and finally to the catholyte. In our model, we characterized the transport of these ions through the boundary layer and the membrane, in a first instance separately and then combined. For all equations, the following notations are used: *C* is the concentration and *f* is the fraction. The first subscript (*i*) represents the ion. The second subscript (*z*) represents the location of the ionic species: 0 is anolyte solution–boundary layer interface, bl is the boundary layer–CEM interface, and m is the CEM–catholyte solution interface. *L*_bl_ is the boundary layer thickness, *L*_m_ is the membrane thickness, and *z* is the dimensionless distance from the anolyte to catholyte, where the ions move.

For the described system, the anode is in the steady state, therefore we can write the NP equation as
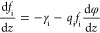
1where *f*_i_ is the ion fraction expressed as the ratio of the total counter-ion concentration (*C*_T,0_); *q*_i_ is the ion charge (in this case, it is always one as only monovalent ions are considered); γ_i_ is the dimensionless transported current of the ion given by (eq 2); and φ is the dimensionless potential expressed as φ = *E*·*F*·*R*·Temp (where *E* is the potential, *R* is the gas constant, Temp is the temperature, and *F* is Faraday’s constant).

### Dimensionless Operational Parameters

Different operational parameters, such as the applied current, can be controlled when membrane electrolysis is used for ion recovery. This current relates to the ions transported through the membrane, and it will determine the effluent concentration. To make the current (γ) dimensionless, we considered the same variables as for the individual fluxes previously described:
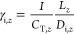
2where *I* is the molar current density (mol·m^–2^·s^–1^) and *D*_i,*z*_ is the ion diffusion coefficient at location *z* (m^2^·s^–1^).

### Ion 1 Transport Number at the Boundary Layer–IEM Ensemble

The ion transport number (*t*_i_) can be derived for the combined boundary layer (bl)–IEM ensemble at the anode side and for the IEM. First, the relationships for the boundary layer and IEM are derived separately, and later these are combined.

#### Boundary Layer, Selectivity, and Limiting Current

As previously stated, in the anolyte, three ions are assumed to be present (1 = NH_4_^+^, 2 = Na^+^, and 3 = Cl^–^), of which the NP equation describes the transport. Considering an ideal selective membrane, we assume that the flux of Cl^–^ through the CEM is zero. This means that the ratio of the flux of total anions and total cations (d*f*_T_/d*z*) can be described as
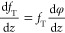
3

Furthermore, we also know that the transported current (γ) equals the total flux of cations through the CEM and that the sum of the fractions of ion 1 and ion 2 is one, therefore
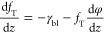
4

Combining eqs 3 and 4 gives a linear solution for the counter-ion concentration and thus also for the sum of ions 1 and 2
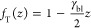
5

As the fraction of total anions (*f*_T_(*z*)) at the anode solution–boundary layer interface is one, we find the first constraint, which is the maximum current density at the boundary layer–membrane interface (γ_bl_ ≤ 2). At the boundary layer, we have both counter and coions flux. As Cl^–^ cannot move through the CEM, it enhances the flux of T^+^ to compensate the gradient between the bulk and the membrane and therefore sets a maximum current for the boundary layer of two (twice the flux of T^+^). This maximum current can be achieved when the counter-ion concentration becomes zero at the boundary layer–membrane interface (*z* = bl). If the membrane allows a certain coion transport (leakage), the transport of counter-ions will slightly decrease.^102^

Using the relationship described in eq 5, we can find the ordinary differential equations for ion 1
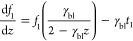
6

From the solution of eq 6 [d’Alembert equation], we can find the fraction of ion 1 at the membrane interface.
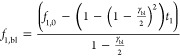
7

As the fraction cannot become negative, at the boundary layer, the following constraint limits the ion transport
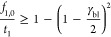
8

Furthermore, this sets a maximum current through the boundary layer that is reached when the transport of ion 1 is higher than its fraction and vice-versa, namely
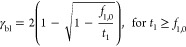
9
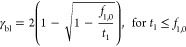
10

The first equation (eq 9) represents the maximum current when the transport number of ion 1 is higher than its fraction in the anolyte. Therefore, there is a limited amount of ion 1. This means that the current can be considered as overlimiting current. There is current available to transport more ions even though the anode solution is depleted.

The second equation (eq 10) represents the situation when the transport of ion 1 is lower than its fraction. This can be considered the ohmic limited region for the counter ion transport.

#### Membrane Selectivity and Current Relationship

We will also investigate the effect of current on the ion transport through the CEM. Additionally, the catholyte of an ES for ammonia recovery has such a pH (pH ≫ 10) that the concentration of NH_4_^+^ can be considered zero.

We assumed an ideal CEM and anolyte composition with only monovalent ions. At the catholyte–membrane interface, the total fractions of all ions equal the fractions in the solution. However, inside the CEM, the total concentration of the ionic species equals the fixed charge of the IEM. We also know that the sum of the fractions of all cations equals one (*f*_1_ + *f*_2_ = 1). The NP equation for both ions 1 and 2 thus gives
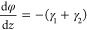
11

Because all ions considered are monovalent, the sum of the ionic fluxes equals the current in the membrane (γ_m_). When adding this to eq 1, we thus find
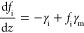
12

The solution of this equation and the resulting ion transport are
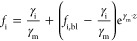
13

Mind that γ_i_/γ_m_ is the transport number of i (current efficiency of the desired ion). From eq 12, we can calculate the transport number as both the concentration on the anode side (*f*_i_,_bl_) and the concentration on the cathode side (*f*_i_,_m_) of the membrane are known as well as the applied current density. The transport number (*t*_i_) is presented in eq 14
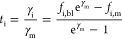
14

If we consider the transport obtained in eq 14, the fraction of ion 2 in the membrane (*f*_2,m_) is related to the fraction of ion 2 in the boundary layer (*f*_2,bl_)
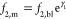
15

However, the sum of the counter-ion fractions is maximally one, which sets a maximum current through the membrane (γ_m,max_). This means that when the fraction of ion 2 at the cathode is near one, the ion 1 that can be transported toward the cathode is still fraction dependent. This maximum current in the membrane allowing a steady state equals
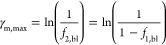
16

This means that when the current at the membrane is below the maximum current, the transport number of ion 1 is one (*t*_1_ = 1 for γ_m_ ≤ γ_m,max_). For currents higher than γ_m,max_ and assuming ion 1 is entirely extracted from the cathode (*f*_1,m_ = 0), we then find a new relation for *t*_1_
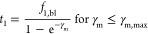
17

#### Boundary Layer–Membrane Ensemble

Overall, we observe that the boundary layer and IEM both present a maximum current (γ_bl_ and γ_m_). This means each layer has its own influence on the ion transport number. So far, we have discussed the boundary layer and membrane selectivity separately, but they both determine the overall ion transport. In the system, the total flux through the boundary layer is the same as the total flux through the CEM. As γ_bl_ has a constant maximum value (eq 9), we will further express the membrane current as a function of the boundary layer current (γ_m_ = αγ_bl_). The value of α represents the ratio between the flux of ions through the membrane and the boundary layer
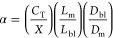
18

From eq 14, it is possible to observe that the selectivity of the boundary layer–membrane ensemble is dependent on (i) the concentration of ions in the membrane solution, (ii) on the thickness of both regions, and (iii) both the diffusion coefficients in the membrane and the boundary layer. By combining eqs 8 and 18, we find that the ion transport number for ion 1 from the anolyte through the ensemble to the catholyte can be described as
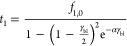
19

**Figure 2:**
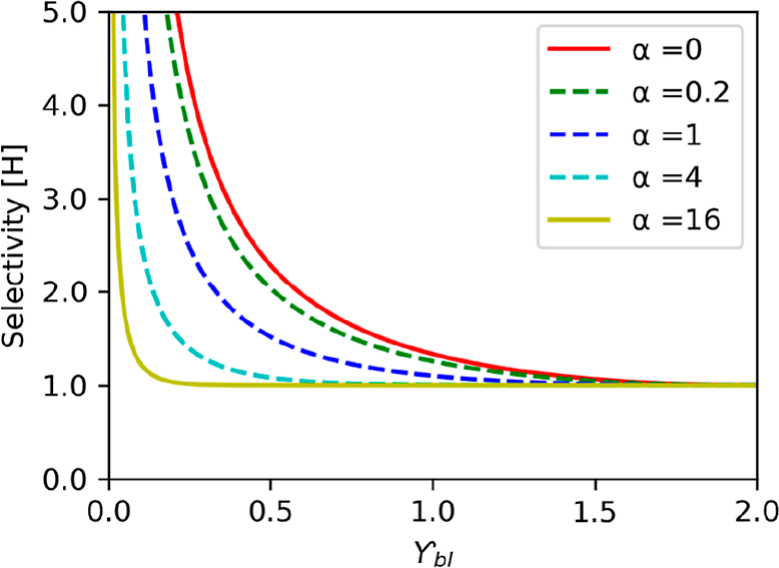
Effect of the boundary layer–membrane properties (represented in α) on the selectivity (*H*) as a function of the current (γ) (eq 20). Different α were considered. With the current increase, the selectivity tends to one.

Here, we see that the transport number is directly proportional to the fraction, while the denominator accounts for the effects of current and the size of the boundary layer. For the ease of notation, we will further write eq 19 as
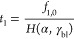
20where *H* is the combined selectivity function of the boundary layer–membrane ensemble. The boundary layer–membrane selectivity shows how an ion (ion 1) is transported preferentially over the other (ion 2). Figure 2 shows the effect of α on selectivity as a function of the current.

Figure 2 shows the lower the current (γ) is, the more selective is the IEM for ion 1. Additionally, it shows a non-selective region for higher currents (*H* is ∼1). Once this current is reached, the physical properties of the IEM and boundary layer do not affect the system, and the fractions influence the transport in the anolyte solution. However, the smaller the α, the higher the current we can operate the system before a reduction of the selectivity occurs.

As the fraction of ions in solution is maximum one, eq 20 is only valid if the fraction of ion 1 in the anolyte is equal to or smaller than the combined selectivity:
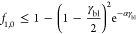
21

For a smaller current (γ ≤ γ_max_), this relationship does not hold, and the transport number of ion 1 equals one, as described in eq 13. The combination of boundary layer and IEM always allows a current, such that γ_bl_ ≤ 2. The ideal α is close to zero. It represents the situation when the selectivity is only affected by the membrane properties, meaning the highest selectivity possible is observed (no current effect).

## Results and Discussion

In practical applications, the main interest is maximizing the removal and membrane transport of the species recovered toward the cathode to improve the extraction process. Using the relationships established above, we will describe how the transport is affected by current and, consequently, how an inverse selectivity is observed at the boundary layer and CEM for certain conditions. The effect of the maximum current on the transport of the recovered ion 1 in the catholyte will be described at different ion fractions in the influent (*f*_1_) for both the boundary layer and CEM. Additionally, a maximum current for the membrane electrolysis system on the different selectivity (α) is established.

The boundary layer acts as a preselective region before the ions cross the membrane.

**Figure 3:**
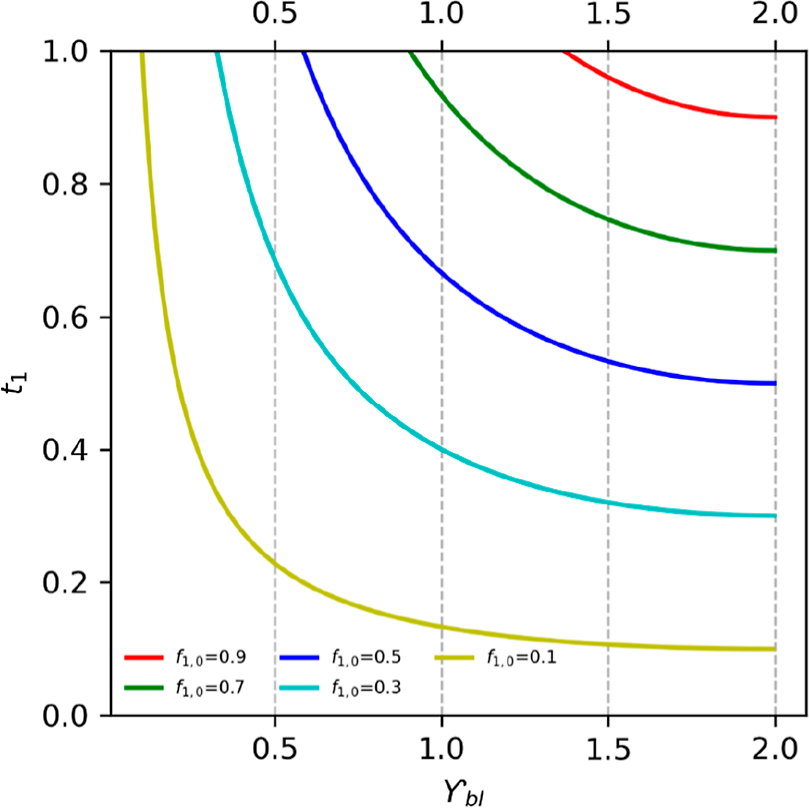
Limiting current (γ_bl_) at the boundary layer as a function of the ion 1 transport number (*t*_1_) in relation to the current was described at different ion fractions in the influent solution. Overall, the ion transport number decreases with the current.

The model established that the studied system has a maximum current in the boundary layer due to the depletion of ions in the anolyte–boundary layer interface. Figure 3 shows γ_bl,max_ (maximum dimensionless current) through the boundary layer expressed as function a of the ion transport number of ion 1 (*t*_1_) for different fractions of ion 1 at the anode (*f*_1,0_). For example, when *f*_1_ is 0.3, 30% in solution is NH_4_^+^ and 70% is Na^+^.

When the transport number of ion 1 equals its fraction in the influent solution, the system can be operated at the highest current, see Figure 3.

As ion 1 is recovered, we are especially interested in those situations, where the transport number is higher than the fraction (*t*_1_> *f*_1_) at the anolyte solution. This would mean that a higher removal/extraction of ion 1 could be achieved independent of its fraction in solution (the system is more selective). The maximum current is smaller due to either ion 1 or ion 2 depletion in all other cases. This means the boundary layer limits the ions in the boundary–layer membrane interface (*L*_bl_) and consequently acts as a selective region itself.

The maximum current shifts according to the fraction of ion 1 in solution (eq 9). A high transport number of ion 1 can only be observed at currents lower than this maximum current when the fraction of ion 1 is higher than its transport. Thus, a maximum flux of ion 1 is determined by the fraction of ion 1 in the anolyte (*f*_1,0_) and the maximum current.

In the membrane, the exclusive transport (*t*_1_ = 1) of ion 1 only occurs at low current densities.

**Figure 4:**
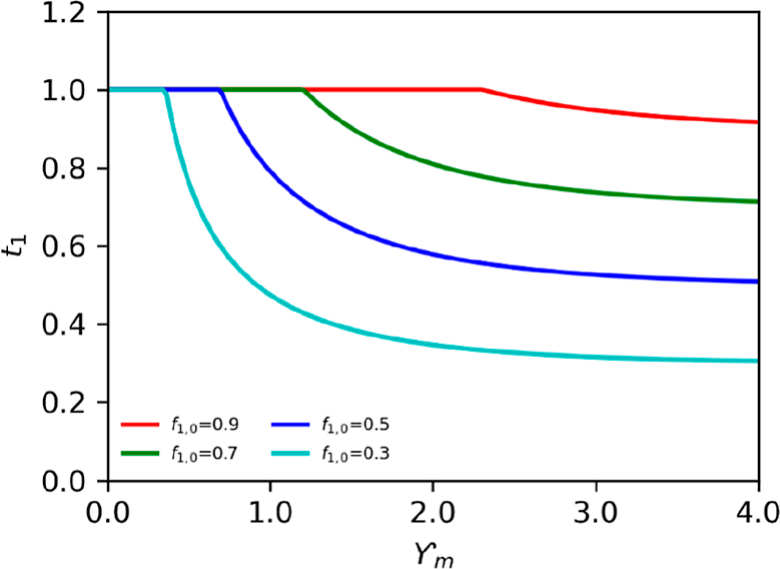
Ion 1 transport number as a function of the current (γ_m_) through the CEM at different ion fractions in the influent solution. Initially, the ion 1 transport number is equal to one. Once we reach a certain current, the transport number decreases. The higher the ion 1 fraction, the higher the currents we can reach.

Equation 17 was used to calculate the transport number of the ions through the membrane. Figure 4 shows the transport number as a function of the current through the membrane.

Figure 4 shows that the exclusive transport of ion 1 (*t*_1_ = 1) over the CEM only occurs when its fraction is lower than the transport number and at low currents, as the transport is both a function of current and fraction. When current increases, the maximum transport number achieved equals the fraction of ion 1 in the anolyte solution (*f*_1,0_). Here, we are in a limiting current regime for ion 1, and transport of ion 2 occurs.

The transport of cations over the CEM is limited by the conditions obtained at the membrane interfaces with both anolyte and catholyte. We assumed an ideal CEM, where no anions are transported. However, the fraction of ion 2 (*f*_2,2_) in the catholyte is close to one, while the fraction of ion 1 (*f*_1,2_) is assumed to be equal to zero as it is removed/extracted from the cathode. This means that the ion 2 concentration gradient over the membrane increases while the ion 1 concentration decreases. When a current lower than the maximum current is used, the transport of Na^+^ is slowed down by an increase of the concentration gradient in the membrane. This results in selective transport of ion 1. However, the effect is diminished when the current increases. When we operate the system at a current higher than the maximum membrane current (γ_m,max_), we will always observe both the transport of ion 1 and ion 2. The accumulation of ion 2 in the cathode creates a concentration gradient over the membrane that opposes the electric field strength. However, the ion fraction can be maximally one and sets the maximum current through the membrane (γ_m,max_).

Steady state in the catholyte would only occur when the transport number of ion 2 is zero (meaning no Na^+^ would be transported) and when the fraction of ion 2 at the cathode side equals the fraction at the boundary layer (γ_m_ = 0) (eq 15). However, in many practical examples, a flux of both ion 1 and ion 2 toward the cathode is observed.^21,22,34−36^ Meaning most studies are operating above limiting current.

Membrane electrolysis paired with an extraction process should be operated well below the limiting current density.

In addition to the boundary–layer membrane ensemble effect on the selectivity, a maximum current can be established to achieve a certain fraction at the anolyte, while the ion 1 transport number is one.

**Figure 5:**
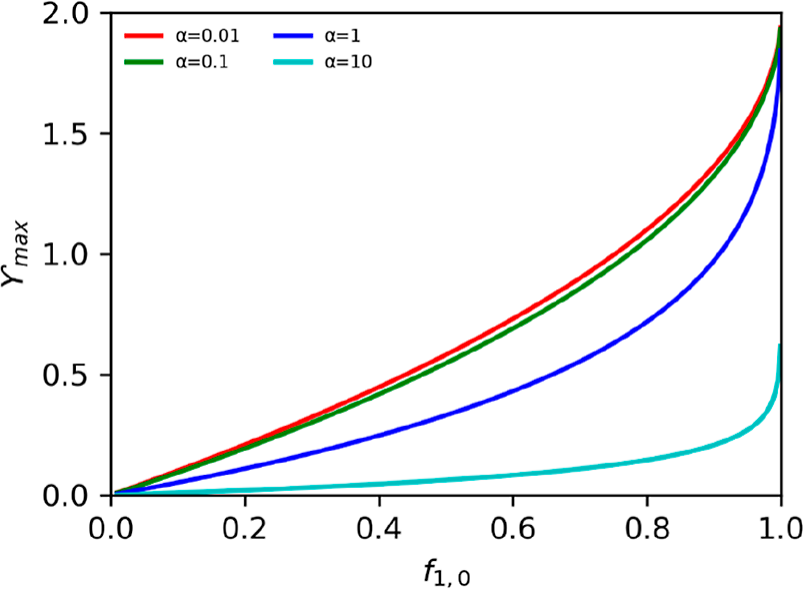
Maximum current (γ_max_) the ES can operate at different fractions of ion 1 in the anolyte, while maintaining exclusive transport of ion 1 (*t*_1_ = 1). The influence was characterized for different α. When the solution is depleted, no current can be applied (zero current, zero fraction). The higher the fraction in the anolyte, the higher the current the system can be operated.

Figure 5 shows the maximum current the system can be operated to achieve a certain ion 1 fraction at different boundary–layer membrane property ratios (α), while the transport of ion 1 over the membrane equals one. First, the maximum current of the system should be decreased to match the fraction of ion 1 in the anolyte. Furthermore, removing ion 1 entirely from the anode is more difficult as the system becomes ion depleted at the boundary layer for lower fractions, particularly when α is further from ideal (α > 0).

At low current, the boundary layer and membrane ensemble is extremely selective as only ion 1 is transported (*t*_1_ = 1). When we increase the current, we observe that the double layer–membrane ensemble selectivity (*H*, eq 20) plays a role, and the transport number of ion 1 as it decreases with increasing current.

The equation below gives the maximum current
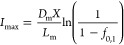
22

Considering the IEM data compiled by Veerman,^37,38^ where for a Nafion CEM, the fixed charge (*X*) for a CEM is 4 mol L^–1^; the *D*_m_ is 5 × 10^–12^; and an average membrane thickness is 1 × 10^–4^ m, we can calculate the maximum current density. From eq 2 and assuming 70% of the cations in solution is ammonium (as found in urine and reject water^39,40^), the maximum current density is approximately 23 A m^–2^. Although a higher limiting current value of the IEM was previously quantified (>200 A m^–2^),^38,41^ the exclusive transport of NH_4_^+^ (*t*_1_ = 1) can only be achieved at a current density below 23 A m^–2^.

When it comes to the boundary layer versus membrane properties included in the parameter α, the ideal case is α close to zero. Here, the system can achieve the highest transport for ion 1 at the maximum limiting current, meaning a selective IEM with infinite high conductivity (e.g., by reducing the membrane thickness) and/or a boundary layer with thickness close to zero (e.g., by increasing the liquid recirculation speed). Besides the physical characteristics of the membrane, α is influenced by the thickness and diffusivity of the boundary layer, parameters that are affected, for example, by the recirculation speed of the solution in the compartment^9,42^ or by the ion concentration in the system (a characteristic of the wastewater).^29,43^

The model provides a simplified description for all membrane electrolysis, where a dynamic cathode is imposed by the extraction of one of its species. What we often see in practical examples is that several cationic species are transported over the CEM when operating an electrochemical system for nutrient recovery.^10,22,44^ If only one ion is of interest, ideally, we would like to have a transport number of one (*t*_1_ = 1) as this would mean a high recovery and high current efficiency. However, the transport of a single species over the CEM is rarely reached. Here, while only considering the Nernst–Planck equation to describe the fluxes, we see that this can be the result of a competing ion 2 and the fact that the ion 1 is constantly removed from the catholyte. In order to maintain the flux of ion 1, we need the flux of ion 2, therefore, *t*_1_ is never equal to one. We need to keep the flux of Na^+^ to keep a certain flux of NH_4_^+^. Consequently, this flux is related to a maximum current. In equilibrium, a constant removal of ions occurs from the anolyte (constant composition) through the CEM, but the catholyte concentrations are variable (a steady state is never reached). At present, most electrochemical recovery processes are performed at higher current densities (>20 A·m^–2^), therefore observe the transport of all cationic species, meaning an inefficient use of energy.^25,45^ Ammonium is often the most transported charge (around 60%) over the cation exchange membrane. If sufficient current is applied, the ammonium transport often matches its fraction in wastewater such as urine or rejects water (60–70%).^21,22,25,35^

## Conclusions

Using the Nernst–Planck equation, we predicted that during electrochemical ion recovery, the transport of a single ionic species of interest through an IEM could only be achieved at low current densities. Once the maximum current is surpassed, the concentration gradient formed over the membrane results in the transport of the different species from the anolyte solution. When operating an ES, we should consider both the properties of the IEM/boundary layer (here described as α) as well as the selectivity of both regions.

## List of acronyms

α: ratio between the flux of ions through the membrane and the boundary layer
bl: boundary layer
*C*: concentration
CEM: cation exchange membrane
*D*_i,z_: ion diffusion coefficient
*E*: potential
ES: electrochemical system
*F*: Faraday’s constant
*f*_i_: ion fraction
γ: dimensionless current
*H*: selectivity
*I*: molar current density
IEM: ion exchange membrane
*L*: thickness
m: membrane
NP: Nernst–Planck
φ: dimensionless potential
*q*_i_: ion charge
*R*: gas constant
*T*^+^: total cations
*T*^–^: total anions
*t*_i_: ion transport number
Temp: temperature
*z*: dimensionless distance from anolyte to catholyte where the ions move
